# Minimally invasive plate osteosynthesis (MIPO) for mid-shaft fracture of the tibia (AO/OTA classification 42): A retrospective study

**DOI:** 10.1016/j.amsu.2020.11.033

**Published:** 2020-11-11

**Authors:** Hyunseong Kang, Jung-Kook Song, Joseph Y. Rho, Jaehwang Lee, Jaewon Choi, Sungwook Choi

**Affiliations:** a Department of Orthopedic Surgery, Jeju National University School of Medicine, Jeju, South Korea; b Department of Preventive Medicine, Jeju National University School of Medicine, Jeju, South Korea

**Keywords:** Tibia, Mid-shaft fracture, Minimally invasive plate osteosynthesis

## Abstract

**Background:**

There is abundance of literature regarding the treatment of tibial mid-shaft fracture, and intramedullary nailing (IMN) is described as the treatment of choice. However, problems such as malunion and knee pain are known disadvantages of this approach. Minimally invasive plate osteosynthesis (MIPO) technique is another treatment option for tibial mid-shaft fracture.

The purpose of this study is to evaluate the clinical, radiological results, and complication rates of tibial mid-shaft fractures treated with MIPO technique.

**Materials and method:**

Thirty-seven skeletally mature patients who underwent MIPO for a mid-shaft fracture of tibia (AO/OTA classification 42) from June 2016 to May 2018 were retrospectively reviewed. A total of 37 patients (12 females, 25 males) with a mean age of 52.7 years (range 28–78 years) were included. The clinical and radiological outcomes, such as the Jeju Lower Extremity Trauma Scale (JLETS), time to callus formation, time to bony union, and complications such as delayed union, malunion, nonunion, and infection were assessed.

**Results:**

Bony union was achieved in all cases but one (36 cases). Average callus formation was observed in 10.7 (6.5–14.5) weeks. The average time to union was 19.8 (11.5–26.5) weeks. The average JLETS score was 46.9 (40–53) point. Malunion deformities were observed in 3 cases (8.1%). Two superficial infection cases all resolved spontaneously. There was no statistically significant difference in clinical and radiographic outcomes by different AO/OTA fracture types.

**Conclusion:**

The MIPO technique with locking compression plate provides stable fixation and satisfactory clinical and radiological results for mid-shaft fractures of tibia irrespective of the fracture type. Future study should aim to compare MIPO and IMN cases directly to clarify the differences and similarities between the two treatment modalities.

## Highlights

• Intramedullary nail is regarded as golden standard for tibial diaphyseal fractures.
• Knee pain and instability are some known disadvantages of intramedullary nails.
• Minimally invasive plating can avoid many drawbacks of intramedullary nailing.
• Minimally invasive plating is effective regardless of fracture types.

## 1 Introduction

Tibial shaft fractures are among the most difficult injuries to treat due to the precarious blood supply and the relatively low amount of soft tissue covering the tibia, which often leads to open fracture, severe complications and major disabilities. Common issues in the treatment of tibial shaft fractures are nonunion, malunion, and infection.

There is abundance of literature regarding the treatment of diaphyseal tibia fracture, and intramedullary nailing (IMN) is described as the treatment of choice for majority of the cases. It promotes biological bone healing with preservation of osteogenic fracture hematoma while providing mechanical stability [1]. However, problems such as malunion with rotational, axial malalignment and knee pain are known disadvantages of this approach [2,3]. Especially in distal and proximal tibia shaft fractures, intramedullary nailing is reported to be associated with malalignment issues much more commonly than minimally invasive plate osteosynthesis (MIPO) with locking compression plate (LCP) technique [[4], [5], [6]].

As mentioned earlier, MIPO technique is another treatment option for tibial shaft fracture. It also provides good preservation of blood supply and fracture hematoma at the fracture site, promoting secondary biological bone healing. For distal tibial fractures, MIPO technique is shown to provide reliable fixation by achieving anatomical reduction and restoring the alignment of the limb, thereby allowing early rehabilitation exercise and ambulation. Wound problems were frequent before the MIPO technique became popular, but with the advent of the technique, they are much less reported nowadays as MIPO technique spares subcutaneous soft tissue of anterior medial tibia and enables adequate soft tissue coverage overlying the plate [5]. Minimally invasive surgery has been described widely for proximal and distal tibial fractures, especially for fractures of the metadiaphyseal and peri-articular areas. The outcomes of this technique showed good wound healing as well as fracture union [[7], [8], [9], [10]].

As described, MIPO technique is widely used for proximal and distal tibial fractures presently. However, there was a paucity of literature describing the use of MIPO technique for diaphyseal tibial mid-shaft fractures (AO/OTA classification 42). As the indications for LCP fixation in tibial fractures were traditionally emphasized in osteoporotic, metadiaphyseal and comminuted peri-articular fractures [1], there was a need to examine its usefulness in the treatment of diaphyseal, mid-shaft fractures as well. We hypothesized that there would be no difference in postoperative clinical and radiological outcomes between different fracture types treated with MIPO, and that they would be comparable in efficacy compared to IMN. This paper was designed to provide one of the first insights on the treatment of mid-shaft tibial fractures with MIPO to widen surgeon's choice, and to deepen our understanding of tibial fractures. In this retrospective study, we reviewed our experience and results in treating tibia mid-shaft fractures using the MIPO technique in 37 patients in order to assess the differences in the clinical, radiological results and complication rates between different types of the fracture.

## 2 Materials and Methods

### 2.1 Patient selection

This study was approved by the Institutional Review Board at our institution (IBR # 2015-08-014), and was in accordance with the 1964 Helsinki declaration and its later amendments or comparable ethical standards. The study is registered with the Research Registry and the unique identifying number is: researchregistry6194. The work has also been reported in line with the STROCSS criteria [11].

We retrospectively analyzed the patients who had undergone procedures using MIPO technique for mid-shaft fractures of the tibia (AO/OTA classification 42) between June 2016 and May 2018. A total of 37 patients (12 females, 25 males) with a mean age of 52.7 years (range 28–78 years) were included. Table 1 presents the demographic characteristics.

**Table 1:** General characteristics of the patients (N = 37).

Patient Parameter	Value
**Mean age, (years)**	52.7 ± 14.3 (28–78)
**Sex, n (%)**	
**Male**	25 (67.6)
**Female**	12 (32.4)
**Smoking, n (%)**	8 (21.6)
**Diabetes, n (%)**	12 (32.4)
**AO/OTA**^a^**classification, n (%)**	
**42-A**	9 (24.3)
**42-B**	15 (40.5)
**42-C**	13 (35.1)

a Orthopaedic Trauma Association.

Tibial shaft fractures were categorized according to the Orthopaedic Trauma Association Classification (AO/OTA) scheme based on the initial anteroposterior and lateral plain radiographs of the lower leg, and then 3D reconstructed computerized tomography (CT) scan images were taken for surgical planning [12].

Skeletally mature patients with extra-articular fracture of the tibia (AO/OTA classification 42) were included in the study. The tibial mid-shaft was defined as only the diaphyseal shaft excluding the meta-diaphyseal junction area. Patients with intra-articular tibial fractures (AO/OTA classification 41 or 43), pathologic fractures, neurovascular injury, and who were younger than 17 years of age (skeletal immaturity) or unsuitable for rehabilitation were excluded.

### 2.2 Clinical and radiological evaluation

Operative time, delayed surgery (and number of days when applicable), time to callus formation, time to bony union, post-operative complications, and clinical performance were assessed by reviewing medical records. Time to bony union and callus formation were evaluated by periodic radiography. Patients were followed up at four, twelve and twenty-four weeks, and one and two years post-operatively. They revisited the outpatient clinic and anteroposterior (AP) and lateral plain x-rays of their tibia had been obtained.

Both the immediate post-operative and the final follow-up radiographs were compared to assess the accuracy of reduction and final alignment. Measurements were performed for coronal (varus and valgus) and sagittal (procurvatum and recurvatum) plane deformities using the measuring technique described by Freedman and Johnson [13].

In AP view, varus/valgus deformity was evaluated by measuring the angle between the lines drawn perpendicular to the proximal and distal tibial articular surfaces. In lateral view, the procurvatum/recurvatum deformity was measured similarly and 7° of posterior slope was subtracted.

Delayed union was defined as radiographic union after three months, while nonunion was defined as lack of any healing within six months. Malunion was defined as coronal deformity (varus or valgus angulation) of >5°, sagittal deformity (anterior or posterior angulation) of >10°, rotational deformity of >15°, and/or shortening of >2 cm. Rotational alignment, shortening, and knee range of motion (ROM) were assessed clinically. Rotational deformity was measured as foot-thigh angle in the clinic at follow-up visits [[13], [14], [15], [16]].

To assess the functional outcome, we used the Jeju Lower Extremity Trauma Scale (JLETS). The questionnaire examines the following 3 categories: anterior knee pain assessed by the visual analogue scale (VAS) pain scoring system (10 points), activity score (30 points), range of motion (ROM) (10 points), and tenderness of the fractured site (5 points) at sixth postoperative month. With respect to VAS pain scoring, the JLETS scoring system assigned 10 points for VAS 0 to 1, 8 for 2 to 3, 6 for 4 to 5, 4 for 6 to 7, 2 for 8 to 9, and 0 for 10. Overall, JLETS is a 55-point scale, and a higher score represents a better clinical outcome (Table 2) [17]. We evaluate all lower extremity trauma patients at our institution using the JLETS scoring at their routine postoperative follow-up visits.

**Table 2:** Jeju lower extremity trauma Scale (JLETS).

Subject	Range	Point
**I. Pain: VAS**^a^**score (10 points)**	0–1	10
	2–3	8
	4–5	6
	6–7	4
	8–9	2
	10	0

**II. Activity Score (30 points)** Scale of difficulty None = 3 points Mild = 2 points Moderate = 1 points Extremely = 0 point	Standing	0–3
	Walking	0–3
	Ascending stairs	0–3
	Descending stairs	0–3
	Running	0–3
	Sitting	0–3
	Rising from sitting	0–3
	Rising from bed	0–3
	Bending to floor	0–3
	Heavy domestic duties	0–3

**III. Range of motion (10 points)** Flexion contracture affected joint	<5°	10
	5°–9°	8
	10°−14°	6
	15°−19°	4
	20°−24°	2
	≥25°	0

**IV. Tenderness at fractured site (5 points)**	No	5
	Yes	0

TOTAL Points		( )/55

a Visual analogue scale.

### 2.3 Surgical technique

All procedures were performed by two trauma-specialized orthopaedic surgeons (S.C. and H.K.) at a university hospital trauma center. All cases received proximal lateral or distal medial Low Bend Locking Compression Plates (LCP) (Synthes, Zuchwil, Switzerland), respectively.

The patients were positioned supine on the radiolucent table with 15° of flexion of the knee joint. Tibia was exposed proximal and distal to the fracture site, and fracture reduction was achieved by indirect reduction techniques with pointed reduction forceps. The skin was incised approximately 2–4 cm distal or proximal to the fracture site depending on the fracture location, and a tunnel was submuscularly made with Cobb's elevator, through which the plate was passed through under fluoroscopic guidance. Following insertion of the plate, correct positioning of the plate was confirmed by fluoroscopy. After placement of locking screws, fluoroscopy was performed again to check the alignment. Additional fixation was achieved with screws at both ends of the plate so at least three screws were placed on each side.

### 2.4 Rehabilitation protocol

Partial weight bearing was allowed and continuous passive motion (CPM) (Artromot K1 Standard, ORMED GmbH, Germany) of knee and ankle were initiated from second postoperative day. The CPM angle was adjusted according to the patients’ tolerability. Full weight bearing was permitted when pain was absent and callus formation was radiologically confirmed.

### 2.5 Statistical analysis

Between-group comparisons were made using the χ2 test (or Fisher's exact test if the expected frequency was <5 in any one cell) for categorical variables, and student's t tests (or the Wilcoxon rank sum test for a variable without normality) for continuous variables. All statistical analyses were performed using SPSS 24.0 (SPSS Inc., Chicago, IL, USA). Significance was set at *p* < 0.05.

## 3 Results

A total of 37 patients were included in the study, with 9, 15 and 13 for AO/OTA classifications A, B, and C, respectively. (Table 1).

Bony union was achieved in every case but one (36 cases), and it was lost to follow-up. All patients had the plates removed after the first operative year and had been tracked for the duration of two years, except for the one nonunion case. Callus formation was observed at 10.7 (6.5–14.5) weeks on average, and the average time to bony union was 19.8 (11.5–26.5) weeks. A few complicated cases with angular/rotational deformity and length shortening were observed in 3 cases (8.1%). Two superficial infection cases all resolved spontaneously.

Various operative outcomes were compared between the three groups (AO/OTA classification 42-A, B, and C). (Table 3).

**Table 3:** Comparison of variables across groups.

Patient Parameter	Group A (N = 9)	Group B (N = 15)	Group C (N = 13)	*p*-value
**Age, n (%)**				0.331
**< 45 years**	6 (66.7%)	4 (26.7%)	6 (46.2%)	
**45**–**64 years**	2 (22.2%)	5 (33.3%)	5 (38.5%)	
**> 65 years**	1 (11.1%)	6 (40.0%)	2 (15.4%)	
**Sex, n (%)**				0.547
**Male**	5 (55.6%)	10 (66.7%)	10 (76.9%)	
**Female**	4 (44.4%)	5 (33.3%)	3 (23.1%)	
**Smoking, n (%)**	4 (50.0%)	4 (50.0%)	0 (0.0%)	***0.028***
**Diabetes, n (%)**	1 (11.1%)	4 (26.7%)	2 (15.4%)	0.657
**Delay to operation, n (%)**	8 (24.2%)	12 (36.4%)	13 (39.4%)	0.270
**Delayed days to operation (days)**	3.3 ± 4.5	5.1 ± 4.4	9.2 ± 5.0	***0.016***
**Operative time (minutes)**	113.7 ± 52.6	112.1 ± 30.8	130.4 ± 51.5	0.487
**Time to callus formation (weeks)**	11.6 ± 3.1	16.0 ± 7.6	15.7 ± 8.4	0.300
**Time to bony union (weeks)**	29.3 ± 16.4	31.7 ± 13.7	29.6 ± 14.5	0.898
**JLETS**^a^	46.1 ± 3.4	47.2 ± 4.0	47.0 ± 2.8	0.727
**Visual analogue scale**	8.7 ± 1.0	8.7 ± 1.0	9.1 ± 1.0	0.529
**Activity**	26.3 ± 1.2	26.7 ± 1.3	26.5 ± 1.5	0.847
**Range of motion**	8.9 ± 1.1	8.9 ± 1.0	9.1 ± 1.0	0.957
**Tenderness**	2.2 ± 2.6	3.0 ± 2.5	2.3 ± 2.6	0.818
**Complication, n (%)**				
**Delayed union**	1 (11.1%)	6 (40.0%)	6 (46.2%)	0.257
**Nonunion**	1 (11.1%)	0 (0.0%)	0 (0.0%)	0.243
**Malunion**	1 (11.1%)	1 (6.7%)	1 (7.7%)	1.000
**Infection**	0 (0.0%)	1 (6.7%)	1 (7.7%)	1.000

a Jeju Lower Extremity Trauma Scale.

No significant difference in preoperative demographic characteristics such as sex and age was found between the three groups. Moreover, operative time between the groups was comparable also. However, average delayed days to surgery were 3.3, 5.1, and 9.2 days for each group and the difference was statistically significant (*p* = 0.016). Callus formation and time to bony union on radiograph were comparable among the groups as well (*p* = 0.300, 0.898, respectively).

The average JLETS scores were 46.1, 47.2, and 47.0 for AO/OTA classification A, B, and C, respectively, and they were statistically insignificant (*p* = 0.727). JLETS components – Pain score, Activity, ROM, and tenderness – failed to show statistical significance as well (*p* = 0.529, 0.847, 0.957, and 0.818, respectively).

Lastly, incidence of delayed union, nonunion, malunion and infection between the groups were comparable (*p* = 0.257, 0.243, 0.200, 1.000).

## 4 Discussion

The study investigated various postoperative outcomes in patients with tibial mid-shaft fractures treated with the MIPO technique. We found that there were no significant differences in the rate of postoperative complications such as infection, malunion, delayed and nonunion, and clinical performance assessed by the JLETS score regardless of the fracture classification.

Previous studies demonstrated that complex and/or segmental tibial shaft fractures are associated with longer healing time and possible complications such as malunion [18,19]. This was expected to be seen in our results as well, but there was no observed correlation between fracture type and union complications.

The strength of our study is that it sought to investigate diaphyseal tibia fracture cases treated with the MIPO technique, which is a study design truly difficult to find. Diaphyseal tibia fractures have long been considered best treated with IMN due to its advantages such as minimal soft tissue dissection, good bone union rate and early return to daily living. However, as mentioned earlier, there are specific clinical situations where IMN is inappropriate. Also, anterior knee pain is reported as a complication following as high as 86% of IMN in various studies [20,21]. A study showed that nail tip placement within proximal third from plateau to tibial tuberosity, and having more than 5 mm of nail prominence from anterior tibial cortex were factors related to knee pain after IMN of diaphyseal fractures of the tibia [22]. Other studies have sought to find the cause of knee pain from the surgical approach, nerve transection, violation of the fat pad or joint capsule, and the nail diameter, but it is still very much unclear [23]. This ambiguous, undesirable pain can be avoided as a whole with the MIPO technique. In our study, the patients reported average JLETS pain score of 8.8 (range 8–10). None of the patients reported debilitating pain from the operation that prevented them from activities of daily living. Unlike postoperative pain arising from IMN, the patients did not complain about anterior knee pain, but rather the pain was more localized to sites of incision. Moreover, malalignment has also been one of major drawbacks of IMN, especially in fractures near the knee or ankle joints [4]. IMN is also frequently reported to result in malunion more commonly than MIPO technique in extra-articular distal tibia fractures [24]. All in all, aforementioned shortcomings of IMN can possibly make it desirable for the surgeon to choose the MIPO technique over IMN in diaphyseal tibia fractures.

According to a meta-analysis by Hattarki et al. [25], mid-shaft tibial fractures treated with IMN took 23.3 weeks (range 16–36) on average for union, while 25% of the cases developed malunion, 10% nonunion, and 20% superficial infection. The results of our study were comparable, taking 19.8 weeks (range 11.5–26.5) on average for union, and 8.1%, 2.8%, and 5.6% of the cases developing malunion, nonunion, and superficial infection, respectively. Although a direct conclusion may not be drawn from this observation, it suggests that MIPO yields similar results to IMN in the treatment of Imid-shaft tibial fractures.

MIPO technique has shown excellent clinical outcomes for periarticular, proximal and distal tibial shaft fractures. On the contrary, studies on MIPO fixation of the diaphyseal mid-shaft are rare, primarily because IMN is the historically established, stable means of treatment. However, there are situations where MIPO has clear advantages over IMN. A cadaveric study on nine specimens has shown a novel posteromedial MIPO approach for diaphyseal tibia fracture that can be used as an alternative to IMN in patients with, for example, poor anterior and medial soft tissue condition, peri-implant or periprosthetic fractures, open physis, and blocked access to intramedullary canal [26].

At the end of the day, the clinical and radiological results of our study portray that MIPO technique yields acceptable postoperative outcomes in the treatment of diaphyseal tibia fractures irrespective of the type of fracture.

There are limitations to this study. First, a much larger study directly incorporating cases treated with IMN should be conducted in the future to see if MIPO is as effective a treatment as IMN in diaphyseal fractures. If so, the surgeon will have the freedom to choose whichever method as he seems fit. Because our study did not compare the results with that of IMN, it cannot be concluded whether MIPO technique is superior, worse, or similar to IMN in the treatment of diaphyseal tibia fractures. Second, two surgeons with varying clinical experience have participated in the study, and it may have affected the integrity of the data.

## 5 Conclusion

Since intramedullary nailing is not without complications or disadvantages and MIPO technique is capable of reducing iatrogenic soft tissue injury and damage to the bone vascularity and preserving the osteogenic fracture hematoma resulting in good to excellent outcomes, MIPO technique is a reliable approach towards tibial mid-shaft fractures that are not suitable for intramedullary nailing. Soft tissue complications, misalignment and knee irritation problems are avoided.

The use of LCP with MIPO technique may give good results for bone union as well as soft tissue healing. The smaller surgical wounds also lessen the pain around the operation site and thus accelerate postoperative rehabilitation and hasten recovery. As shown, the MIPO technique with LCP provides stable fixation and satisfactory clinical and radiological results for mid-shaft fractures of tibia irrespective of the fracture type.

## Ethical approval

All procedures performed in studies involving human participants were in accordance with the ethical standards of the institutional and/or national research committee and with the 1964 Helsinki declaration and its later amendments or comparable ethical standards. As the following study was performed in retrospectively, formal consent was not required.

## Funding

This research was supported by the 2020 scientific promotion program funded by Jeju National University.

## Author contribution

Sungwook Choi: participated in designing the research, and drafting manuscript.

Hyunseong Kang: participated in designing the research, and statistical analysis.

Jung-Kook Song: participated in statistical analysis.

Joseph Y. Rho: participated in collecting data, writing assistance and proof reading.

Jaehwang Lee: participated in writing manuscript.

Jaewon Choi: participated in writing manuscript.

## Consent

The design and protocol of this study were approved by the institutional review board at our hospital, and every patient filed a written consent.

## Registration of research studies

Name of the registry: Research Registry‬‬‬‬‬‬‬‬‬‬‬‬

Unique Identifying number or registration ID: researchregistry6194.

Hyperlink to your specific registration (must be publicly accessible and will be checked): https://www.researchregistry.com/register-now#home/registrationdetails/5f9e171e2c76cb0015bd2706/

## Provenance and peer review

Not commissioned, externally peer-reviewed.

## Guarantor

Sungwook Choi, M.D., Ph.D.

## Declaration of competing interest

No benefits in any form have been received or will be received from a commercial party related directly or indirectly to the subject of this article.

## References

[1] Anuar-Ramdhan I., Azahari I. (2014). Minimally invasive plate osteosynthesis with conventional compression plate for diaphyseal tibia fracture. Malayasian Orthop. J..

[2] Casstevens C., Le T., Archdeacon M.T., Wyrick J.D. (2012). Management of extra-articular fractures of the distal tibia: intramedullary nailing versus plate fixation. JAAOS-J. Am. Acad. Orthop. Surg..

[3] Lai T.C., Fleming J.J. (2018). Minimally invasive plate osteosynthesis for distal tibia fractures. Clin. Podiatr. Med. Surg..

[4] Vallier H.A., Cureton B.A., Patterson B.M. (2011). Randomized, prospective comparison of plate versus intramedullary nail fixation for distal tibia shaft fractures. J. Orthop. Trauma.

[5] Liu X.k., Xu W.n., Xue Q.y., Liang Q.w. (2019). Intramedullary nailing versus minimally invasive plate osteosynthesis for distal tibial fractures: a systematic review and meta‐analysis. Orthop. Surg..

[6] Pairon P., Ossendorf C., Kuhn S., Hofmann A., Rommens P. (2015). Intramedullary nailing after external fixation of the femur and tibia: a review of advantages and limits. Eur. J. Trauma Emerg. Surg..

[7] Claes L., Heitemeyer U., Krischak G., Braun H., Hierholzer G. (1999). Fixation technique influences osteogenesis of comminuted fractures. Clin. Orthop. Relat. Res..

[8] Helfet D.L., Suk M. (2004). Minimally invasive percutaneous plate osteosynthesis of fractures of the distal tibia. Instr. Course Lect..

[9] Kao F.C., Tu Y.K., Su J.Y., Hsu K.Y., Wu C.H., Chou M.C. (2009). Treatment of distal femoral fracture by minimally invasive percutaneous plate osteosynthesis: comparison between the dynamic condylar screw and the less invasive stabilization system. J. Trauma Acute Care Surg..

[10] Krackhardt T., Dilger J., Flesch I., Höntzsch D., Eingartner C., Weise K. (2005). Fractures of the distal tibia treated with closed reduction and minimally invasive plating. Arch. Orthop. Trauma Surg..

[11] Agha R., Abdall-Razak A., Crossley E., Dowlut N., Iosifidis C., Mathew G. (2019). STROCSS 2019 Guideline: strengthening the reporting of cohort studies in surgery. Int. J. Surg..

[12] Marsh J. (2007). Fracture and dislocation classification compendium-2007: orthopaedic trauma classification, data-base and outcome committee. J. Orthop. Trauma.

[13] Freedman E.L., Johnson E.E. (1995). Radiographic analysis of tibial fracture malalignment following intramedullary nailing. Clin. Orthop. Relat. Res..

[14] Milner S. (1997). A more accurate method of measurement of angulation after fractures of the tibia. J. Bone Joint Surg. Br. Vol..

[15] Puloski S., Romano C., Buckley R., Powell J. (2004). Rotational malalignment of the tibia following reamed intramedullary nail fixation. J. Orthop. Trauma.

[16] Li Y., Jiang X., Guo Q., Zhu L., Ye T., Chen A. (2014). Treatment of distal tibial shaft fractures by three different surgical methods: a randomized, prospective study. Int. Orthop..

[17] Choi S., Lee T.J., Kim S., Cho C., Shim S., Kang H. (2019). Minimally Invasive Plate Osteosynthesis (MIPO) technique for complex tibial shaft fracture. Acta Orthop. Belg..

[18] Mundi R., Axelrod D., Heels-Ansdell D., Chaudhry H., Ayeni O.R., Petrisor B. (2020). Nonunion in patients with tibial shaft fractures: is early physical status associated with fracture healing?. Cureus.

[19] Teraa M., Blokhuis T.J., Tang L., Leenen L.P. (2013). Segmental tibial fractures: an infrequent but demanding injury. Clin. Orthop. Relat. Res..

[20] Gustilo T., Shaw A. (1997). Knee pain after intramedullary tibial nailing: its incidence, etiology, and outcome. J. Orthop. Trauma.

[21] Toivanen J.A., Väistö O., Kannus P., Latvala K., Honkonen S.E., Järvinen M.J. (2002). Anterior knee pain after intramedullary nailing of fractures of the tibial shaft: a prospective, randomized study comparing two different nail-insertion techniques. JBJS.

[22] Soraganvi P., Anand-Kumar B., Rajagopalakrishnan R., Praveen-Kumar B. (2016). Anterior knee pain after tibial intra-medullary nailing: is it predictable?. Malayasian Orthop. J..

[23] Cazzato G., Saccomanno M., Noia G., Masci G., Peruzzi M., Marinangeli M. (2018). Intramedullary nailing of tibial shaft fractures in the semi-extended position using a suprapatellar approach: a retrospective case series. Injury.

[24] Li B., Yang Y., Jiang L.-S. (2015). Plate fixation versus intramedullary nailing for displaced extra-articular distal tibia fractures: a system review. Eur. J. Orthop. Surg. Traumatol..

[25] Hattarki R., Bhagat S., Patel K., Singh B., Mehendiratta D. (2016). Comparative study between intramedullary interlock nailing and plating in distal metaphyseal fractures of tibia. Surg. Curr. Res..

[26] Wajnsztejn A., Pires R.E.S., Dos Santos A.L.G., Labronici P.J., Fernandes H.J.A., Ferretti M. (2017). Minimally invasive posteromedial percutaneous plate osteosynthesis for diaphyseal tibial fractures: technique description. Injury.

